# Reliability of Educational Content Videos in YouTube^TM^ about Stainless Steel Crowns

**DOI:** 10.3390/children9040571

**Published:** 2022-04-17

**Authors:** Prabhadevi C. Maganur, Zaki Hakami, Ravi Gummaraju Raghunath, Sudhakar Vundavalli, Ganesh Jeevanandan, Yousef M. Almugla, Sanjeev B. Khanagar, Satish Vishwanathaiah

**Affiliations:** 1 Department of Preventive Dental Sciences, Division of Pediatric Dentistry, College of Dentistry, Jazan University, Jazan 45142, Saudi Arabia; 2 Department of Preventive Dental Sciences, Division of Orthodontics, College of Dentistry, Jazan University, Jazan 45142, Saudi Arabia; dr.zhakami@gmail.com; 3 Department of Preventive Dental Sciences, Division of Pediatric Dentistry, College of Dentistry, King Faisal University, Al-Ahsa 31982, Saudi Arabia; gummarajuravi@gmail.com; 4 Department of Preventive Dentistry, Division of Community Dentistry, College of Dentistry, Jouf University, Sakaka 42421, Saudi Arabia; dr.sudhakar.vundavalli@jodent.org; 5 Department of Pediatric and Preventive Dentistry, College of Saveetha Dental, Saveetha Institute of Medical and Technical Sciences, Saveetha University, Chennai 602105, India; helloganz@gmail.com; 6 Department of Preventive Dental Sciences, Division of Orthodontics, College of Dentistry, King Faisal University, Al Ahsa 31982, Saudi Arabia; yalmugla@kfu.edu.sa; 7 Preventive Dental Science Department, College of Dentistry, King Saud Bin Abdulaziz University for Health Sciences, Riyadh 11481, Saudi Arabia; sanjeev.khanagar76@gmail.com; 8 King Abdullah International Medical Research Center, Riyadh 11481, Saudi Arabia

**Keywords:** video information and quality index, tooth preparation, space maintainer, pediatric dentistry, stainless steel crown

## Abstract

The purpose of the present study is to evaluate the quality and educational information provided on YouTube^TM^ about stainless steel crowns (SSC). Videos were searched for on YouTube ^TM^ using keywords related to stainless steel crowns in pediatric dentistry in the Google Trends application. A total of 52 videos were available. After exclusion criteria application, 22 videos were selected for the study for analysis. To classify the video content as high or low quality, a scoring system formed of seven parameters was used. For a global evaluation of the video quality, the video information and quality index were applied. Data obtained were analyzed statistically. Only a few videos explained the importance of SSCs. Most of the videos uploaded with a high number of likes were of low quality. Very few videos were of high quality. The content quality and educational quality of videos is poor and incomplete. None of the videos explained the need for the SSC and its benefits in pediatric dentistry.

## 1. Introduction

In the current era of digitalization, access to information from various social platforms is much easier and faster than before. Factors that trigger the use of the internet to pursue medical information include a patient’s desire for greater access to information, quick and convenient accessibility, and an affordable method for professional healthcare consultations [1]. Video-sharing websites are among the most established information sources, and of these, YouTube^TM^ is the most popular website, generating more than 2 billion views every day [2]. A video is uploaded to YouTube^TM^ every minute, and an average user spends at least 15 min on the platform daily. Although the scientific authenticity of these videos is contentious [3], YouTube^TM^ is one of the most frequented platforms by both casual and professional users [4].

In terms of the number of site visits, the highest traffic goes to Google, Facebook, and YouTube^TM^, respectively [5], and research suggests that 80% of internet users search the internet to access healthcare information [6]. Social media enables patients to easily seek and exchange information and knowledge preceding and following treatments. However, the quality of this online information is not guaranteed; therefore, it can affect users’ and professional students’ understandings by misleading them. In light of this, studies have been undertaken to determine the quality of the information presented by YouTube^TM^ videos on dentistry [5,7,8], medicine [9,10,11], and other health-related subjects.

Stainless steel crowns (SSC) represent an extremely durable dental restoration with many clear-cut indications for primary tooth applications. They are used following a pulpectomy/pulpotomy for teeth with developmental defects or large carious lesions that involve multiple surfaces, and they are used when amalgam fails, for individuals with high caries risk, and for fractured teeth. Studies that have investigated the longevity of restorations by comparing the lifespan and reliability of SSCs and Class II amalgams have demonstrated the superiority of SSCs [12].

According to our research, no previous study has assessed the quality of the information provided by YouTube^TM^ videos about SSCs. Such a complex subject requires visual information to explain certain concepts to parents and patients, and, as previously described, with more people using audio/video media sites daily, new videos are uploaded and regularly updated by experts. Therefore, it is important that patients’ and physicians/dentists’ knowledge is constantly updated. To ensure this, the quality of available data needs to be compared with previous studies.

This study’s objective was to determine the quality of the information presented by YouTube^TM^ videos for patients seeking information about SSCs. We also aimed to understand this platform to ensure that patients and dentists are kept up to date with contemporary knowledge.

## 2. Methods

### 2.1. Study Design

This study used a cross-sectional methodology.

### 2.2. Search Strategy

We searched for YouTube^TM^ videos uploaded up until October 2021 using the search term ‘stainless steel crown’. Google Trends indicated that this was a common term. Search engines also indicated the use of keywords such as SSC and stainless-steel crowns. This information was used to analyze video with the following conditions.

### 2.3. Inclusion and Exclusion Criteria

The inclusion criteria were as follows:

- Video quality of 240p or more was deemed acceptable
- Only videos in the English language were considered
- The primary video content was set as stainless steel crown

The exclusion criteria were as follows:

- Repeated videos were discarded
- Videos without sound and explanation were discarded
- Videos in any language other than English were discarded

### 2.4. Content Evaluation

One of the researchers (P.C.M) carefully checked and evaluated all the videos. Prior to the assessment, intra-observer calibration determined a Kappa value of 0.92. The video content was evaluated according to the following criteria: (i) Explaining the rationale behind SSCs, (ii) Advantages, (iii) Limitations, (iv) Indications, (v) Contraindications, (vi) Procedure (LA, isolation, crown selection, tooth preparation, crown adaptation, finishing and polishing, occlusal adjustments, cementation), and (vii) Postoperative instructions.

Scoring criteria

The content criteria listed above were used to scale each video, with each element scoring 1 point out of a possible total score of 15 points. Videos with a total content score of less than or equal to 7 were categorised as low-quality content, while videos with a total content score higher than or equal to 8 were categorised as acceptable content.

VIQI Index assessment: [13,14]

The video information and quality index (VIQI) were used for evaluating each video’s general quality. The criteria considered during assessment for VIQIs include Information Accuracy, Information Flow, Quality (addition of images, video headings, animation, public members’ reports, and summary), and Sensitivity (the degree of compatibility between the title and content of the video) of videos. The video information was categorized in terms of high-quality (5 score) and low-quality (1 score) using the Likert-type scale.

General parameter evaluation

After reviewing, all the videos were split into five fundamental groups, referring to the individual who had uploaded them to the Web: (1) Dental clinic/university, (2) Dentist/specialist, (3) Commercial company, (4) Layperson, and (5) Others

One of the researchers (P.C.M) reviewed each video and noted certain general parameters which were taken into consideration. The parameters which were considered for evaluation were Running Time, Sum of Likes, Sum of Dislikes, Time Since the Video was Uploaded, Number of Views and Comments.

Video power index (VPI): [13,15]

The video’s popularity was evaluated in terms of the following formulas.

Like ratio: The following formula was calculated to show the interaction with users in terms of likes/dislikes and number of views.

The following formulas were used to evaluate viewer’s interaction.

$$\frac{Number\;of\;likes+number\;of\;dislikes}{Number\;of\;views}\times 100$$

View ratio

$$\frac{Number\;of\;views}{Number\;ofdays\;since\;the\;video\;was\;uploaded}\times 100$$

The scores for the general parameters and the content parameters were collected and evaluated to identify any correlation. Any doubts in scoring or categorizing the videos were resolved by discussing and reviewing the footage with S.V., R.R., G.J., and S.A.A, specialised in pediatric dentistry, until a consensus was reached.

It was not required to obtain approval from the ethics committee since this study worked with publicly available data, and also anonymity of videos was maintained.

Statistical analysis

The IBM SPSS version 20 software was used to analyze the data. The Kolmogorov–Smirnov test was initially employed to evaluate the normality of data (content scores). The content quality score was compared with multiple parameters with the Spearman’s correlation, whereas the Man–Whitney U test was employed for comparison of the dichotomous variable (i.e., acceptable or low content). A *p*-value typically ≤0.05 was considered statistically significant.

## 3. Results

The final analysis included 22 YouTube^TM^ videos (Table 1). We determined that paediatric dentistry specialists uploaded 59.1% of the videos, educational universities uploaded 22.7% of the videos, and commercial organisations uploaded less than 10% of the videos. The instructor’s name was not specified in two of the videos. Of all the videos, 14 used a patient for the demonstration, and 8 used a typhodont model for the demonstrations.

The characteristics of the videos are presented in Table 1. The median (i.e., interquartile range) length of the videos was 3.8 (1.2–9.5) min. The median number of views was 2738 (560.5–21,466.2), the median number of likes was 9 (9–65.5), and there were no dislikes. The median number of subscriptions was 234 (42.7–44,144.2), and the median number of days since the videos were uploaded was 1199 (408.5–1685.2). The median VPI score was 2.2 (0.67–8.4), the median content score was 3 (1–7.8), and the median VIQI score was 4 (4.0–16.0).

Spearman’s correlation was used to correlate the content score with various parameters; statistically significant correlations was found for Time elapsed since upload date (days), Length of video (min), Number of likes, Number of views, VPI score, and all components of VIQI (Table 2).

As per the content scores, videos with low content scores and acceptable content scores were divided. This dichotomous variable was compared with multiple parameters by the Mann–Whitney U test. Statistically, a considerable association was observed between the number of views, the number of likes, high-content video, the quality component of the VIQI score, total VIQI score, and VPI score (Table 3).

## 4. Discussion

Patients are increasingly using the internet to make informed healthcare decisions, comprehend their existing medical condition, and seek appropriate treatments [16]. YouTube^TM^ has become increasingly popular among dental students and patients. It has a wide variety of health-related information [17]. Typically, at the beginning of their course professional students prefer audiovisual content over a scientific platform. As YouTube^TM^ can be accessed from any place at any time, it is the most popular education tool, including in the field of life sciences [18].

Social media platforms make it easy for patients and their parents to communicate as well to share their views. However, there are also some risks that can occur when personal experiences are being shared. Misinformation is caused by such inaccurate information when propagated in the society. Social media platforms such as YouTube^TM^ do not go through peer reviews. The quality of the information disseminated by YouTube^TM^ videos is controversial both in the field of medicine and in dentistry [19]. The inaccurate, insufficient, and misleading content of videos on YouTube^TM^ can affect the learning abilities of the student. On the other hand, YouTube^TM^ does not charge anything and is a potent tool for receiving and delivering any form of knowledge to any target audience. On the flipside, this platform is also known for videos that come from dubious sources. As a consequence, non-specialists and dental professionals do not have any means to verify the accuracy of the content [20]. Users on YouTube^TM^ have the authorization for uploading video clips, irrespective of their qualifications, professionalism, and educational background. According to multiple studies, when non-specialists upload videos, their content is likely to include patients’ experiences. As a result, they can propagate incorrect information, especially when compared to videos uploaded by specialists [20,21].^.^

Studies on YouTube^TM^ videos have been documented in the literature related to space maintainers and oral habits. SSCs are one of the gold-standard and primary components in pediatric dentistry. They are utilized for restoring the normal automation/anatomy of the primary dentition before exfoliation. Hence, the dental students or patients’ guardians develop interest to seek information about the subject on YouTube^TM^, owing to its popularity. In our study, around 50 videos were screened, of which only 22 videos could be included for the evaluation. All others were excluded as they were either mute videos, included no explanation of the procedure, or the video was explained as PowerPoint slides. Although specialists uploaded most of the videos, the overall evaluation of the video content was low.

Previously, a wide range of scoring systems was employed to determine video quality of contents. These include GQS (Global Quality Score), JAMA (Journal of American Medical Association) [22] usefulness scoring [23], and VIQI [13,14]. In GQS [24,25], the Likert scale was used to rate videos in terms of effectiveness through a 5-point scale. An overall quality rating is determined by collecting quality content subheadings, such as information accuracy, video’s flow, quality, and usefulness [26]. On the contrary, In the VIQI system, the quality content subheadings were evaluated separately and independently from 1 to 5 points, after which the total quality score is calculated. Thus, we chose the VIQI scoring system for this study over GQS.

During content evaluation, we noted that only a few of the videos explained the importance of SSCs; most of the videos concentrated solely on the procedure. Only one video explained the need for SCCs [Indication and contraindications]. None of the videos provided any post-operative instructions or advice on when to revisit the dentist, despite being uploaded by the specialist in some cases. Also, most of the videos’ title and the explanation of the content did not match.

In contrast to Nilufer et al.’s study [17], we found a correlation between quality assessment methods and the VPI, similar to Aydin and Akyol’s [22] study. We also found that videos uploaded around 5 years ago were low quality but had a high number of likes. The contrast between videos with low content and acceptable content was determined in terms of VPI score, total VIQI, number of views, and number of likes. There were only five high-quality videos with a significant number of likes (*p* = 0.007).

The VIQI, a global evaluation index for video quality showed a significant difference for quality (*p* = 0.033); therefore, it is important for determining the quality of the video contents.

When comparing the durations of the videos, there was no statistical difference between the high-quality and low-quality videos. In contrast, Lena and Dindaroğlu [10]. found a relationship between high-quality content and duration. In the current study, the correlation between the VPI and VIQI assessment methods was significant compared with acceptable and low-content videos. All the high-quality videos were uploaded by specialists (5 out of 13), and they had high correlations with likes, views, VIQI scores, and VPI scores. All five high-quality videos had been uploaded within the last 5 years. The remaining 17 videos were low-quality content. One video with a high number of likes and views was evaluated as low-quality content, and this was uploaded almost 10 years ago.

### Limitations

The limitation of the current study is that it was highly dependent on the chosen keyword. We minimised this by using the most popular term, ‘stainless steel crown’, as determined by Google Trends. Limiting the video content language to English restricted the reviewed content and limited the results by ignoring the popularity of this topic in developing countries, where English is not the native language.

## 5. Conclusions

A wide variety of information has been offered by YouTube^TM^ videos on SSCs. This study investigated the educational quality of these videos, and found it to be incomplete and poor. Most of the videos showed the procedure, but there was no information about why SSCs are required or their purpose. Hence, it is difficult for students and patients seeking information about SSCs and their benefits to learn about the subject. Therefore, professional societies, specialists, and academic institutions should decide how to develop a content standard by offering sources for such mediums. Hence, we recommend that scientific bodies monitor and regularise the content of videos uploaded on social media.

## Figures and Tables

**Table 1:** Characteristics of YouTube^TM^ videos (n = 22).

Characteristic	Mean ± SD ^1^	Median (IQR) ^2^
Length of video (min)	7.6 ± 10.3	3.8 (1.29.5)
Number of views	62,155.2 ± 236,072	2738 (560.5–21,466.2)
Number of subscriptions	58,981.5 ± 124,706.1	234 (42.7–44,144.2)
Number of likes	59.5 ± 101.2	9 (9–65.5)
Number of dislikes	4.7 ± 9.6	0 (0–3.7
Number of comments	2.4 ± 3.6	11.00 (0–4.5)
Time elapsed since upload (days)	1289 ± 1046	1199 (408.5–1685.2)
VPI ^3^	21.5 ± 70.5	2.2 (0.67–8.4)
Content score (15 pts)	4.09 ± 3.8	3 (1–7.8)
VIQI ^4^ score (20 pts)	8.36 ± 6.7	4 (4.0–16.0)
Flow of information	2.27 ± 1.9	1 (1–5.0)
Information accuracy	2.27 ± 1.9	1 (1–5.0)
Quality	1.72 ±1.5	1 (1.0–1.0)
Precision	2.09 ± 1.8	1 (1–5.0)
**Video category**	**n (%)**	
-Education	7 (31.8)	
-People and Blogs	10 (45.5)	
-Science and Technology	4 (18.2)	
-Film and Animation	1 (4.5)	
**Source of Video**		
-University	5 (22.7)	
-Specialist	13 (59.1)	
-Commercial	2 (9.1)	
-Others	2 (9.1)	

^1^ SD: standard deviation. ^2^ IQR: Inter-Quartile Range; ^3^ VPI: Video power index: ^4^ VIQI: Video information and quality index.

**Table 2:** Correlation between content quality score with various parameters.

	Correlation Coefficient	*p*-Value
Time elapsed since upload date (days)	0.542	0.009 **
Length of video (min)	0.441	0.040 *
Number of likes	0.609	0.003 **
Number of dislikes	0.151	0.502
Number of Views	0.481	0.023 *
Number of comments	0.167	0.457
VIQI ^1^ score	0.879	0.000 ***
Flow of information	0.859	0.000 ***
Information accuracy	0.859	0.000 ***
Quality	0.766	0.000 ***
Precision	0.793	0.000 ***
VPI ^2^ score	0.511	0.015 *

* *p* ≤ 0.05, ** *p* ≤ 0.01, and *** *p* ≤ 0.001. ^1^ VIQI: Video information and quality index; ^2^ VPI: Video power index.

**Table 3:** Variable’s comparison according to low (≤8) and acceptable (≥9) content scores.

	Man-Whitney Score	Z-Score	*p*-Value
Time elapsed since upload date (days)	27.000	−0.766	0.484
Length of video (min)	26.000	−0.851	0.434
Number of likes	6.500	−2.520	0.007 **
Number of dislikes	17.500	−1.722	0.118
Number of Views	9.000	−2.298	0.019 **
Number of comments	25.500	−0.979	0.386
VIQI ^1^ score	12.500	−2.430	0.042 *
Flow of information	17.000	−2.003	0.118
Information accuracy	17.000	−2.003	0.118
Quality	11.000	−3.182	0.033 *
Precision	15.000	−2.315	0.081
VPI ^2^ score	8.000	−2.390	0.014 *

* *p* ≤ 0.05, and ** *p* ≤ 0.01; ^1^ VIQI: video interpretation and quality index; ^2^ VPI: video power index.

## Data Availability

The data will be provided upon reasonable request.
